# Supplementary material for: Trichinella spiralis cathepsin L damages the tight junctions of intestinal epithelial cells and mediates larval invasion
Source: PLoS Negl Trop Dis. 2023 Dec 4;17(12):e0011816. doi: 10.1371/journal.pntd.0011816 (PMC10721182; doi:10.1371/journal.pntd.0011816)
Supplement: Attachment — Submitted filename: Response to comments on PNTD-D-23-00702.docx [file pntd.0011816.s001.docx]

**Response to comments on PNTD-D-23-00702**

> The reviewers commented that this is an interesting study, however, editing and potentially other experiments are required before the manuscript is suitable for publication. Please ensure that sufficient information is provided for the methods and address all the comments raised by the reviewers.

-Thank you for your comments and recommendations!

**> Reviewer's Responses to Questions**

**> Reviewer #1:** Methods, line 136. What is the anti-rTsASP2 serum mentioned? Define this and how it was produced.

- Thanks for your comments. Sorry for not describing it clearly! The sentences have been amended as follows in the revised MS.

-“Mouse anti-rTsACatL2 serum, anti-rTsASP2 serum and anti-MBP serum were produced by immunizing BALB/c mice with rTsCatL/rTsASP2/MBP mixed with adjuvant via intramuscular injection and collecting the blood of the mice to isolate the serum [6,14]. ”

> For Western blotting, how much soluble lysate was added per well? How was this quantified?

-Thanks for your comments. A BCA assay kit measured the protein concentration before adding 20 µg of protein to each well for Western blotting. The sentences have been amended as follows in the revised MS.
- “Protein concentration was determined using the BCA assay kit (Beyotime, China).”

- “20 µg” has been added in line 183 of the clean revised manuscript.

> Figure 1 – how many biological replicates of the RNAi experiment have been performed? This should be clearly stated in the text/legend.

-Thanks for your comments. Three biological replicates were performed for the RNAi experiment. The sentences have been amended as follows in the revised MS.
- “Each experiment was done in triplicate.”

> How was the % protein expression (Figure 1) quantified? More detail should be added to line 175 in the methods section.

-Thanks for your comments. The sentences have been amended as follows in the revised MS.

-“And then, the relative intensities of each band were analyzed using the Image J software (National Institutes of Health, USA).”

**> Reviewer #1:** What time point post-RNAi is shown in Figure 2? Please add.

- Thanks for your comments. Figure 2 shows RNAi at 1 day post-RNAi, revised in the revised MS.

> It would be of interest to see if the cellular damage shown in Figure 3 is due only to the mechanical action of worms migrating through the monolayer as whether secreted products contribute as well. Could this assay be performed with the addition of dead worms as well as T. spiralis secreted products and recombinant TsCatL2?

- Thanks for your comments and suggestions. We added the dead worms to the cell surface . Some dead worms were seen on the cell surface, but the worms did not invade the Caco-2 cells and there were no worm tracks on the cell surface (Fig 3 A4). Fig 3 has been replaced with a new Figure as follows.

**Fig 3. Caco-2 cells damage caused by larval penetration.** A: Observation of larval invasion cell. The larva invaded Caco-2 cells (A1, the red arrow shows migratory trace); non-invaded larvae on Caco-2 (A2) and C2C12 (A3); the dead larvae on Caco-2 (A4); Scale bar: 200 μm. B: Larval invasion resulting in cell damage. B1 shows Caco-2 cells monolayer damaged by helminth invasion, stained with PI to show the red fluorescence of the cells; B2 represents undamaged Caco-2 cell monolayer; B3 shows undamaged C2C12 cell monolayer; scale bar: 200 μm. C: Fluorescence analysis of larval migrationin Caco-2 cells. After the larvae have invaded the cell monolayer, some worm proteins remain within the cells. Anti-rTsCatL2 serum and *T. spiralis* infection serum fluoresce green in association with worm proteins; nuclei of damaged or dead cells are stained red with PI; scale bar: 100 μm.

-As Song et al. have already discovered, the cellular barrier is disrupted when *T. spiralis* excretion secretion proteins are added to the surface of Caco-2 cell monolayers (Song et al., 2022). Therefore, *T. spiralis* excretion secretion proteins were not added to this study.

Song YY, Lu QQ, Han LL, Yan SW, Zhang XZ, et al. (2022) Proteases secreted by *Trichinella spiralis* intestinal infective larvae damage the junctions of the intestinal epithelial cell monolayer and mediate larval invasion. Vet. Res. 53: 19.

-In this study, it was discovered that the Caco-2 cell barrier was severely damaged and some of the cells were detached from the six-well plates when rTsCatL2 was added to the liquid medium and co-incubated with Caco-2 cells for 1 h (Figure 6). We did not add rTsCatL2 to the semi-solid medium for the in vitro *T. spiralis* invasion assay because this assay requires observation of the rate of worm invasion after two hours of incubation.

> Additionally, experiments with heat-denatured enzyme (or enzyme inhibited with E-64) would show whether these effects are due to active enzyme (same for later experiments; e.g. Fig 7 and the pulldown in Fig. 9).

- It’s a good comment and suggestion. We agree with you. We supplemented the rTsCatL2+E-64 group and the heat-denatured rTsCatL2 group. Results from immunofluorescence demonstrated that there was no significant change in the morphology of Caco-2 cells after incubation with rTsCatL2+E-64 / heat-denatured rTsCatL2 for 1h, and there was no change in the cellular localization of claudin 1, occludin, zo-1, e-cadherin, or laminin. The results suggest that rTsCatL2 enzyme activity is linked to the disruption of the cellular barrier. Figures 6 and 7 have been replaced with new figures as follows.

**Fig 6. rTsCatL2 binding to Caco-2 cells analyzed by fluorescence.** After co-culture of rTsCatL2 with Caco-2 cells, immunofluorescence showed that rTsCatL2 initially bound mainly around the cell periphery and gradually entered the cytoplasm with increasing incubation time. Faint fluorescence of Caco-2 cells was detected in the MBP and E64-treated rTsCatL2 groups, while no fluorescence was detected in the heat-treated rTsCatL2 group and the PBS group. Scale bar: 50 μm.

**Fig 7. Fluorescence analysis of changes in rTsCatL2 after incubation with Caco-2 cells on cell junctions (A) and extracellular matrix proteins (B).** Immunofluorescence showed that claudin 1, e-cadherin, occludin and zo-1 gradually entered the cells from the cell junctions, the fluorescence brightness of claudin 1 gradually decreased, laminin was scattered in the nucleus, while the localization of cellular proteins did not change in the MBP, PBS, E64-treated rTsCatL2, and heat-treated rTsCatL2 groups, scale bar: 50 μm.

> Figure 4 - I don’t think it is necessary to plot the data as both & % invasion and % inhibition. Pick one readout for this figure.

- Thanks for your suggestion. In the revised version, we have removed the intrusion rate and retained the inhibition rate data. Figure 4 has been replaced with a new Figure as follows.

**Fig 4. The effect of TsCatL in the larval invasion of Caco-2 cells.** A : Larval invasion inhibition by anti-rTsCatL2 immune serum. After adding anti-rTsCatL2 serum at 1:50, 1:100 and 1:200 ratios, invasion was reduced by 39.87%, 36.07% and 33. 33% compared to PBS (*χ^2^*_1:50_ = 65.86，*P* < 0.001；*χ^2^*_1:100_ = 7.334，*P* < 0.05；*χ^2^*_1:200_ = 8.160，**P* < 0.05). B: Effect of RNAi silencing of TsCatL on worm invasion. RNAi silencing of TsCatL inhibited worm invasion into Caco-2 cells, and the rate of worm invasion was lower in the siRNA-487 group (**P* < 0.05).

> How many individual works were measured in each group (Figure 5)?

- Thanks for your comments. Figure 5 measured the length of 20 worms for each group.

> Results describing Figure 6. It is stated in text that “Immunofluorescence with anti-rTsCatL2 antibody showed that rTsCatL2 bound mainly to the tight junctions of Caco-2 cells…”. It is not possible to say for certain what the TsCatL2 is binding to at this resolution. At best you can say green fluorescence was seen around the cell periphery. This section should be tempered to reflect this.

- Thanks for your comments. In the revised version, we have changed the description of the results as follows.

-“Immunofluorescence with anti-rTsCatL2 antibody showed that rTsCatL2 bound mainly around the cell periphery...”

> Collagen is misspelt as “collage” in several places throughout the manuscript.

-Very sorry! The word “Collage” was wrong; it has been revised as “Collagen”

> The labeling of the micrographs shown in Figure 10 are too small/unclear. How many cells/field of view were observed/counted to quantify autophagy at the ultrastructural level?

- Thanks for your comments. The labeling of the micrographs in Figure 10 has been revised. Figure 10 has been replaced with a new Figure. As only three fields of view were taken in each group, they were not analyzed quantitatively.

> **Reviewer #1:** Since the silencing/antibody blocking of TsCatL2 was only partially successful at blocking entry of the worms into the epithelial cells, could the authors please speculate on other enzymes that may be compensating and the implications of this for parasite control.

- Thanks for your comments. *Trichinella spiralis* secretes numerous proteases to aid invading epithelial cells. It has been demonstrated that the *T. spiralis* cathepsin B (TsCB), *T. spiralis* cathepsin L (TsCL) and *T. spiralis* cathepsin X (TsCX) bind to epithelial cells and enhance larval invasion of epithelial cells particularly. Moreover, *T. spiralis* putative serine protease (TsSP), *T. spiralis* aspartic protease-2 (TsASP2), *T. spiralis* galectin (Tsgal) and *T. spiralis* aminopeptidase (TsAP) were involved in larval invasion of host intestinal epithelial cells. A variety of proteases work together to help *T. spiralis* invade epithelial cells.

> **Reviewer #1:** Line 358 – previous research?

-Sorry! The description in the MS has been corrected as follows.

*-*“The *T. spiralis* cathepsin L (TsCatL) protein consists of a transmembrane helix, an inhibitor_I29 domain and a mature Pept_C1 domain. TsCatL two domains (TsCatL2) was cloned and expressed in *Escherichia coli* Rosetta-gami B (DE3).”

**> Reviewer #1:** In this study the authors aimed to investigate the role of Trichinella spiralis cathepsin L (TsCatL2) during penetration of intestinal epithelial cells. Invasion of cells in vitro, as well as establishment of infection of mice, were both reduced when TsCatL2 was silenced using RNAi. They propose a mechanism for this where TsCatL2 disrupts the barrier function of the intestine by degradation of extracellular matrix/cell junction proteins.

- Thank you for your comment!

> Specific comments:

> Introduction, line 106. Replace “substance” with “cysteine peptidase” to describe Giardipain-1 here.

- Thanks for your comments. The word “cysteine peptidase” has been revised as “substance”.

> Introduction, line 109. Explain what domains TsCatL2 refers to – was only the mature enzyme domain cloned and expressed as a recombinant?

- Thanks for your comments. TsCatL2 is an inactive zymogen containing the inhibitor 129 and Pept_C1 domains. The description in the MS has been amended as follows.

“The *T. spiralis* cathepsin L (TsCatL) protein consists of a transmembrane helix, an inhibitor_I29 domain and a mature Pept_C1 domain. TsCatL two domains (TsCatL2) was cloned and expressed in *Escherichia coli* Rosetta-gami B (DE3).”

> Do the authors have any information on the transcriptional profile of TsCatL? Is it specifically upregulated to coincide with arrival of the worms in the intestine? This should be included if known.

- Thanks for your comments. Our previous study found that TsCatL was transcribed and expressed at different developmental stages of T. spiralis, with the highest transcription level in the intestinal infective larvae stage. The description in the MS has been added as follows.

-“It also exhibits the highest transcript levels in the larval stage of intestinal infection”

> Line 376 - it should be made clear that *Fasciola hepatica* secretes a large family of cathepsins L, only some of which (primarily FhCL2 and FhCL3) have collagenolytic activity.

- Thank you for your proposal! The phrase “cathepsin L” has been revised as “cathepsin L2 and cathepsin L3” in revised MS.

> The section of ECM/cell junctions in the discussion was overlong and could be made more concise.

- Thank you for your proposal! The section of ECM/cell junctions in the discussion have been amended as follows in revised MS.

-“In this study, rTsCatL2 altered the localization of cellular tight junction proteins (claudin 1, occludin and zo-1) and adhesion-linked proteins (E-cadherin), downregulated claudin 1 expression and disrupted intestinal barrier integrity. The transmembrane protein claudin 1 is the major TJ protein in the paracellular barrier [42]. The transmembrane protein occludin is a crucial molecule regulating TJ permeability [43]. The cytoplasmic protein zo-1 is a key molecule involved in TJ assembly and signal transduction [44]. Changes in the number and localization of TJ proteins result in reduced intestinal epithelial barrier function [45].”

-“Previous research has demonstrated that rTsCatL2 hydrolyzes host collagen I, fibronectin, and laminin in vitro, all of which are important components of the extracellular matrix (ECM), which is required for cell adhesion, signaling, migration, and metabolism [14,46]. In this study, rTsCatL2 altered the localization of laminin. However, Western blot showed no change in laminin and collagen I expression, presumably because rTsCatL2 interacts with cells for only one hour and rTsCatL2 degrades host proteins in vitro by overnight incubation [14]. Further research into rTsCatL2's interactions with Caco-2 proteins showed that rTsCatL2 interacted with collagen I and laminin but not with claudin 1, E-cadherin, occludin, or fibronectin. The results showed that the cell barrier disruption by rTsCatL2 does not rely solely on its hydrolysis but that there are other regulatory mechanisms.”

> Suggested further experiments are suggested in the results comments.

- Thank you for your suggestion! We have added control experiments as suggested.

**> Reviewer #2:** The methods used are appropriate and the results support the conclusions.

> 1. Line 191,how to obtain "anti-MBP serum" should be describe clearly ;

- Sorry for not describing it clearly! Anti-MBP serum were prepared in our laboratory by immunising BALB/c with MBP-labelled protein. The sentences have been amended as follows in the revised MS.

- “Mouse anti-rTsACatL2 serum, anti-rTsASP2 serum and anti-MBP serum were produced by immunizing BALB/c mice with rTsCatL/rTsASP2/MBP mixed with adjuvant via intramuscular injection and collecting the blood of the mice to isolate the serum [6,14]. ”

> 2. Line 209~211, “rTsCatL2, MBP or PBS were ...... added to the surface of Caco-2 cells”，MBP here is protein-tag, how to obtain MBP? also expressed in E.coli and purified？ or Purchased from the company?

- Thanks for your comments. Sorry for not describing it clearly! MBP was expressed in *Escherichia coli* Rosetta-gami B (DE3) and then purified using starch resin. We have added this in line 141 of the clean revised manuscript.

> 3. The abbreviation that first appears should indicated，such as NBL, EBSS.

-Thanks for your comments. Sorry for the lapse calami! The abbreviations have been revised as follows in revised MS.

- “zonula occludens protein 1 (Zo-1)”

- “mitogen-activated protein kinase (MAPK)”

- “Specific pathogen Free (SPF)”

- “small interfering RNA (siRNA)”

- “glyceraldehyde-3-phosphate dehydrogenase (GAPDH)”

- “propidium iodide (PI)”

- “newborn larvae (NBL)”

- “polyvinylidene fluoride (PVDF)”

- “radio immunoprecipitation assay (RIPA)”

- “earle's balanced salt solution (EBSS)”

- “monodansylcadaverine (MDC)”

- “ultraviolet (UV)”

> **Reviewer #2:** The results are clearly and believable，and several minor problems should be noted.

> 1. Line 321~324，“rTsCatL2 bound mainly to the tight junctions of Caco-2 cells..., no binding of MBP to Caco-2 cells was detected.” actually, Fig 6, MBP-tag showed faint fluorescenec，you should describe more accurately.

> - Thanks for your comments. The sentences have been amended as follows in revised MS.

> “...faint fluorescenec of Caco-2 cells was detected.”

> 2. Fig 8 and Fig 9 seem to be contradictory. Fig 8, the rTsCatL2 was added to Caco-2 cells, western blot showed no change in laminin and collagen I expression; and Fig 9, showed rTsCatL2 did not interact with claudin 1, e-cadherin, occludin and fibronectin but with collagen I and laminin. How to explain?

- Thanks for your comments. At first, we were surprised by this result, but three repetitions of the experiment gave consistent results.

-Collagen I and laminin are essential components of the extracellular matrix. The extracellular matrix provides attachment sites and structural support for cells, participates in intercellular information transfer and regulates cell growth, polarity, shape and migration (Theocharis et al., 2016). Laminins are indispensable building blocks for cellular networks, physically bridging the intracellular and extracellular compartments and transmitting signals critical for cellular behavior (Aumailley et al., 2013). Furthermore, our previous study found that rTsCatL2 hydrolyzed laminin and collagen I by overnight incubation with laminin and collagen I (Liu et al., 2022). In this study, rTsCatL2 only interacted with Caco-2 cell monolayers for 1 hour, which is also not the optimal pH for rTsCatL2 hydrolysis. Therefore, no significant hydrolysis of laminin and collagen I by rTsCatL2 was observed.

Theocharis AD, Skandalis SS, Gialeli C, Karamanos NK. Extracellular matrix structure. Adv Drug Deliv Rev. 2016, 97:4-27.doi: 10.1016/j.addr.2015.11.001.

Aumailley M. The laminin family. Cell Adh Migr. 2013, 7(1):48-55. doi: 10.4161/cam.22826. Epub 2012 Dec 21.

Liu RD, Meng XY, Li CL, Long SR, Cui J, Wang ZQ. Molecular characterization and determination of the biochemical properties of cathepsin L of *Trichinella spiralis*. Vet Res. 2022, 53(1):48. doi: 10.1186/s13567-022-01065-6.

> **Reviewer #2:** Minor revision. The quality of English needs improving, I suggest that you obtain assistance from a colleague whose native language is English.

-Thank you! The text has been checked carefully and revised.

> **Reviewer #2**: The topic is novel and interesting, and the methods used are appropriate. "MBP" I mentioned above is important, for rTsCatL2 were expressed in Escherichia coli, recombinant protein of Prokaryotic expression may contain endotoxins，it should avoid the effect of endotoxins on Caco-2 cell junctions, when you observe the effect of rTsCatL2 on Caco-2 cell junctions.

- Thank you for your comment! We used an endotoxin detection kit to remove any endotoxin that rTsCatL2 may contain. The following sentences have been added to the revised MS Materials and Methods.

“To eliminate endotoxin interference in rTsCatL2, endotoxin removal resin (Thermo Fisher, USA) was used.”

**> Reviewer #3:** The methods are well described.

> Study design could include addition of specific inhibitor such as E64 to rTsCL2 to show that inhibitor-bound cysteine protease does not affect Caco2 cells. Also, why did authors only test one concentration of enzyme - was the effect of the protease titratable?

-It’s a good comment and suggestion. We agree with you. In the revised manuscript, we added the experimental group with the addition of E64 and found that E64 inhibited the disruptive effect of rTsCatL on the Caco2 cell barrier. Figures 6 and 7 have been replaced with new figures as follows.

**Fig 6. rTsCatL2 binding to Caco-2 cells analyzed by fluorescence.** After co-culture of rTsCatL2 with Caco-2 cells, immunofluorescence showed that rTsCatL2 initially bound mainly around the cell periphery and gradually entered the cytoplasm with increasing incubation time. Faint fluorescence of Caco-2 cells was detected in the MBP and E64-treated rTsCatL2 groups, while no fluorescence was detected in the heat-treated rTsCatL2 group and the PBS group. Scale bar: 50 μm.

**Fig 7. Fluorescence analysis of changes in rTsCatL2 after incubation with Caco-2 cells on cell junctions (A) and extracellular matrix proteins (B).** Immunofluorescence showed that claudin 1, e-cadherin, occludin and zo-1 gradually entered the cells from the cell junctions, the fluorescence brightness of claudin 1 gradually decreased, laminin was scattered in the nucleus, while the localization of cellular proteins did not change in the MBP, PBS, E64-treated rTsCatL2, and heat-treated rTsCatL2 groups, scale bar: 50 μm.

-We did not initially investigate the effect of different titers of rTsCatL2 on Caco-2 cells as this was not considered in the initial experimental design. According to the literature, 25 µg/ml of *T*. spiralis muscle larvae excretion secretion proteins reduce TJ protein expression and disrupt Caco-2 cell monolayers (Li et al., 2001), 20 μg/ml *T. spiralis* intestinal infective larvae excretion secretion proteins can disrupt the Caco-2 cell monolayer (Song et al., 2022), 8 µg/ml *Giardia duodenalis* Giardipain-1 causes junctional, barrier and apoptotic damage in epithelial cell monolayers (Ortega-Pierres et al., 2018). Therefore, we used 10 μg/mL rTsCatL2 in our experiments and found it effectively disrupted the Caco-2 cell monolayers; this concentration was a bit manipulative. Thank you for your suggestion. We will take this into account in future experiments.

Li C, Bai X, Liu X, Zhang Y, Liu L, et al. (2021) Disruption of epithelial barrier of Caco-2 cell monolayers by excretory secretory products of *Trichinella spiralis* might be related to serine protease. Front Microbiol. 17;12:634185.

Song YY, Lu QQ, Han LL, Yan SW, Zhang XZ, et al. (2022) Proteases secreted by *Trichinella spiralis* intestinal infective larvae damage the junctions of the intestinal epithelial cell monolayer and mediate larval invasion. Vet. Res. 53: 19.

Ortega-Pierres G, Argüello-García R, Laredo-Cisneros MS, Fonseca-Linán R, Gómez-Mondragón M, et al. (2018) Giardipain-1, a protease secreted by Giardia duodenalis trophozoites, causes junctional, barrier and apoptotic damage in epithelial cell monolayers. Int J Parasitol. 48(8):621-639.

> In experiments where anti-TsCL2 was used to block parasite entry into cells - reduced at low significance - specificity could have been shown by adding rTsCL2 to the medium and demonstrating that this reversed the effect of the enzyme

-Thank you for your suggestion. We did not perform this experiment because adding rTsCatL2 to the liquid medium severely damaged the Caco-2 cell monolayer within 1 hour, and the cells even detached from the culture plate (Fig. 6). As for in vitro *T. spiralis* invasion assay, the invasion rate of worms into Caco-2 cell monolayers was observed after 2 h of incubation, so we did not add rTsCatL2 to the semi-solid medium for in vitro *T. spiralis* invasion assay.

> **Reviewer #3:** The amount of knockdown by iRNA is shown to be specific and significant but is nevertheless low - less than 50% with all iRNAs and for transcripts and protein -and it does not add confidence that parasites with only this reduction would be necessarily damaged and lack viability.

- Thank you for your comment! In the initial design, we designed 3 siRNA segments, but the knockdown of the siRNA was less than 50% in all of them.

> **Reviewer #3**: I feel that most experiments do not achieve strong significance in parasite invasion or infectivity and additional experiments as suggested above would strengthen the data

- Thank you for your suggestion! We have added some control experiments as suggested.

**> Reviewer #3:** The data could be improved by better experimental design and control experiments as suggested above e.g. titrating protease, using specific inhibitors, uisng reverse inhibtion of antibodies.

- Thank you for your suggestion! As suggested, we have added the rTsCatL2+E-64 group and the heat-denatured rTsCatL2 group.

--------------------

**-Thank you very much again for your review and your time!**
