# Supplementary material for: Trichinella spiralis cathepsin L damages the tight junctions of intestinal epithelial cells and mediates larval invasion
Source: PLoS Negl Trop Dis. 2023 Dec 4;17(12):e0011816. doi: 10.1371/journal.pntd.0011816 (PMC10721182; doi:10.1371/journal.pntd.0011816)
Supplement: Attachment — Submitted filename: Response to comments on PNTD-D-23-00702.docx [file pntd.0011816.s002.docx]

**Response to comments on PNTD-D-23-00702**

**> Reviewer #2**: 1. the sequence of TsCatL2 should have signal peptide, when construct pMAL-c2X/TsCatL2 recombinant plasmid, contained the signal peptide ?

- Thanks for your comments. The recombinant pMAL-c2X/TsCatL2 plasmid does not contain a signal peptide and was constructed from the structure.

**> Reviewer #2:** 1. Line 308，“in the TsCatL siRNA-487”， and line 310 "lower in the siRNA-487 group than in the control siRNA...Fig 4B", I'm not find siRNA-487 in the methods, and Fig 4B showed siRNA-302, but the legend is siRNA-487 group, maybe an error?

-Thanks for your comments. We feel sorry for our carelessness. The word “siRNA-487” was wrong; it has been revised as “siRNA-302”

> 2. legend of Fig4, "the rate of worm invasion was lower in the siRNA-487 group", the picture showed inhibition%, be consistent.

-Thanks for your comments. The sentences in the legend of Fig 4 have been amended as follows in the revised MS.

-“B: RNAi silencing of TsCatL inhibited larval invasion into Caco-2 cells. The inhibition rate of the siRNA-302 group was higher than that of the control siRNA group (*χ^2^* = 32.262, *P* < 0.001).”

> 3. Fig 6 and Fig 7, rTsCatL2,MBP, rTsCatL2+E64, incubation with Caco-2 cells within 60min, your response mentioned "because adding rTsCatL2 to the liquid medium severely damaged the Caco-2 cell monolayer within 1 hour", if co-culture of MBP and rTsCatL2+E64 with Caco-2 cells more than 1h, whether the similar appearance ?

-Thanks for your comments. When MBP and rTsCatL2+E64 were co-cultured with Caco-2 cells for 1 hour, there was no comparable situation. We co-cultured MBP and rTsCatL2+E64 with Caco-2 cells for 1 hour 15 minutes and found no damage to the Caco-2 cell monolayer, but we did not follow up with longer observations.

**> Reviewer #2**: rTsCatL2 downregulated claudin 1 expression, but not interacted with claudin 1, what's the possible mechanisms, should further discussed.

-Thanks for your comments. It’s a good comment and suggestion. We have added more discussion as follows in the revised MS.

-“Studies showed that *T. spiralis* excretion/secretion proteins inhibited the expression of tight junction proteins via the MAPK signaling pathway [9]. Piezo1, a mechanosensitive and nonselective cation channel, regulates intestinal epithelial function by affecting the tight junction protein claudin-1 via the ROCK pathway [47]. L-glutamine led to an increased distribution of claudin-1 at plasma membranes in intestinal porcine epithelial cells by activating CaMKK2-AMPK signaling [48]. ”

9. Li C, Bai X, Liu X, Zhang Y, Liu L, et al. (2021) Disruption of epithelial barrier of Caco-2 cell monolayers by excretory secretory products of *Trichinella spiralis* might be related to serine protease. Front Microbiol. 12:634185. http://doi.10.3389/fmicb.2021. 634185 PMID: 33815318

47. Jiang Y, Song J, Xu Y, Liu C, Qian W, et al. (2021) Piezo1 regulates intestinal epithelial function by affecting the tight junction protein claudin-1 via the ROCK pathway. Life Sci. 275:119254. http://doi. [10.1016/j.lfs.2021.119254](https://doi.org/10.1016/j.lfs.2021.119254) PMID: 33636174

48. Wang B, Wu Z, Ji Y, Sun K, Dai Z, et al. (2016) L-glutamine enhances tight junction integrity by activating CaMK kinase 2-AMP-activated protein kinase signaling in intestinal porcine epithelial cells. J Nutr. 146:501-508. http://doi.10.3945/jn.115.224857 PMID: 26865645

--------------------

**-Thank you very much again for your review and your time!**
